# Apurinic/Apyrimidinic Endonuclease 1 Restricts the Internalization of Bacteria Into Human Intestinal Epithelial Cells Through the Inhibition of Rac1

**DOI:** 10.3389/fimmu.2020.553994

**Published:** 2021-02-02

**Authors:** Gerco den Hartog, Lindsay D. Butcher, Amber L. Ablack, Laura A. Pace, Jailal N. G. Ablack, Richard Xiong, Soumita Das, Thaddeus S. Stappenbeck, Lars Eckmann, Peter B. Ernst, Sheila E. Crowe

**Affiliations:** ^1^ Department of Medicine, Division of Gastroenterology, University of California San Diego, La Jolla, CA, United States; ^2^ Department of Medicine, Division of Rheumatology, University of California San Diego, La Jolla, CA, United States; ^3^ Division of Comparative Pathology and Medicine, Department of Pathology, University of California San Diego, La Jolla, CA, United States; ^4^ Division of ImmunoBiology, Washington University, St. Louis, MO, United States; ^5^ Center for Mucosal Immunology, Allergy and Vaccine Development, Department of Pathology, University of California San Diego, La Jolla, CA, United States; ^6^ Department of Immunology, Chiba University, Chiba, Japan

**Keywords:** intestinal epithelial barrier, invasion, internalization, Rac1, *Salmonella* Typhimurium, AIEC LF82 strain, apurinic/apyrimidinic endonuclease 1

## Abstract

Pathogenic intestinal bacteria lead to significant disease in humans. Here we investigated the role of the multifunctional protein, Apurinic/apyrimidinic endonuclease 1 (APE1), in regulating the internalization of bacteria into the intestinal epithelium. Intestinal tumor-cell lines and primary human epithelial cells were infected with *Salmonella enterica* serovar Typhimurium or adherent-invasive *Escherichia coli*. The effects of APE1 inhibition on bacterial internalization, the regulation of Rho GTPase Rac1 as well as the epithelial cell barrier function were assessed. Increased numbers of bacteria were present in APE1-deficient colonic tumor cell lines and primary epithelial cells. Activation of Rac1 was augmented following infection but negatively regulated by APE1. Pharmacological inhibition of Rac1 reversed the increase in intracellular bacteria in APE1-deficient cells whereas overexpression of constitutively active Rac1 augmented the numbers in APE1-competent cells. Enhanced numbers of intracellular bacteria resulted in the loss of barrier function and a delay in its recovery. Our data demonstrate that APE1 inhibits the internalization of invasive bacteria into human intestinal epithelial cells through its ability to negatively regulate Rac1. This activity also protects epithelial cell barrier function.

## Introduction

Food-borne bacterial infections are a major cause of disease that negatively impacts both quality and quantity of life (1). Frequently occurring acute intestinal infections include *Shigella flexneri*, *Salmonella enterica* serovar Typhimurium and *Campylobacter jejuni*. The incidence of these infections has not changed in recent decades and the Centers for Disease Control and Prevention estimate that 48 million people are infected annually resulting in 128,000 hospitalizations and 3,000 deaths in the US alone (2, 3).

Various food-borne bacterial pathogens translocate effector molecules into host cells that facilitate their entry and survival (3, 4). For example, *S.* Typhimurium has a type III secretion system that manipulates host cell signaling to enable its invasion into multiple cell types (5–7). Adherent-invasive *Escherichia coli* (AIEC) can reside in human intestinal cells or the lumen for prolonged periods of time although they enter host cells less efficiently than *S.* Typhimurium (8–10). *S.* Typhimurium can invade the epithelium of the small and large intestine while AIEC is typically isolated from the small intestine (11–13). Invasion of epithelial cells by bacteria is facilitated by the activation Rho GTPases, including Rac1, subsequent to the translocation of effector molecules mediated by the secretion system (14–16). In turn, activation of Rac1 leads to cytoskeleton rearrangements and internalization of the bacteria.

Apurinic/apyrimidinic endonuclease 1 **(**APE1) is a multifunctional protein that plays a central role in regulating innate immunity and host responses in the context of oxidative stress (17). APE1 physically interacts with Rac1 in the gastric epithelium to inhibit Rac1 function and the accumulation of reactive oxygen species (ROS) (18). Although the entry of bacteria into host cells often involves regulation of Rho GTPases such as Rac 1 (14–16), it is unknown if the effects of APE1 on Rac1 that impact the accumulation of ROS would also modify the internalization of *S.* Typhimurium or AIEC in the intestine. The data show that APE1 regulates invasion of intestinal epithelial cells by *S.* Typhimurium and AIEC through the negative regulation of Rac1.

## Materials and Methods

### Bacterial Strains and Quantification

*Salmonella enterica* serovar Typhimurium (SL1344), as well as isogenic mutants ΔSPI1 and ΔSPI2 and a strain of SL1344 expressing RFP (kind gifts from Drs. Olivia Steele-Mortimer NIAID, Rocky Mountain Laboratory, MT, USA and Brett Finlay, University of British Columbia, Vancouver, BC, Canada) (19, 20) were used at MOI 10. Adherent-invasive *Escherichia coli* (AIEC) strains LF82 and LF82 expressing GFP (a gift from Dr. Phil Smith, University of Alabama) (21), EPEC, *Campylobacter jejuni* strain C31 and *E. coli* strain K12 (obtained from ATCC) were used at an MOI of 100. Bacteria were maintained on LB agar and for experiments, grown in LB broth and diluted 1/100 overnight under oxygen limiting conditions.

Bacteria were quantified by culture to evaluate colony forming units (CFU) as previously described (22–24). To evaluate invasion, extracellular bacteria were killed with gentamicin (500 µg/mL) for 90 min at 37°C, followed by low-dose gentamicin (50 µg/mL) for the rest of the experiment. At indicated times, cells in each well were washed with phosphate-buffered saline (PBS) and lysed in 1% Triton X-100 in PBS for 15 min at 37°C, followed by serial dilution and plating onto LB agar plates as described in detail elsewhere (24).

### Cell Culture

Epithelial cell lines were maintained using standard techniques (25, 26). Briefly, T84 cells (ATCC) were maintained in high glucose F12/DMEM containing L-glutamine and 5% FBS. HT-29 cells (ATCC) were maintained in McCoy’s 5A medium supplemented with 10% FBS. Primary intestinal epithelial cells were isolated and maintained according to the procedures developed previously (27, 28). Biopsy specimens were obtained from adult subjects undergoing medically-indicated ileocolonoscopy. Ethical approval was obtained by the IRB of UCSD and all donors provided written informed consent. Briefly, biopsy specimens were minced, treated with collagenase (37° C, 1 h), washed and filtered. Cultures were maintained in Matrigel and medium containing Wnt3a, R-spondin and Noggin, which was refreshed or passaged every 2–3 days. For monolayer experiments, wells were coated with 1/30 Matrigel for 30 min, which was removed immediately before cells were added.

### Genetic Manipulation of Cells

APE1 levels in T84 were suppressed by gene transduction as previously described (29) using shRNA in the pSIREN vector targeting 3’ outside of the open reading frame, grown under puromycin selection (Sigma, 1µg/ml). HT-29 and primary epithelial cells were virally transduced with the same shAPE sequences in the FG12 vector ( (30), Addgene #14884). PCMV5.1 expression plasmid with wt APE1 was used for complementation of APE1 expression. The open reading frame from this plasmid is not targeted by the shAPE strategy as described elsewhere (31). Briefly, the U6 promoter-APE shRNA cassette from pSIREN was cloned into FG12 which was digested with XbaI and XhoI. Luciferase shRNA was cloned into FG12 and used as a control in these studies as previously described (18, 32).

HT-29 cells were distributed in 24 wells plate or 10 cm dish and transfected with 0.75 µg or 3 µg of control vector DNA or DNA expressing APE1 (using pcDNA 3.0 (33)**)** or Rac1 (Active Rac1 V12, kind gift from Dr. James Casanova University of Virginia, Charlottesville, VA, USA) with 3 µl or 20 µl of Lipofectamine 2000 (Life Technologies) respectively in opti-MEM.

### Antibodies

Antibodies used were: mouse anti-APE1 clone 13B8E5C2 (Novus Biologicals), mouse anti-Rac1 clone 28A (Millipore) and mouse anti-α-Tubulin clone DM1A (Abcam), anti-rabbit or anti-mouse HRP-conjugated IgG (Cell Signaling Technology) and AlexaFluor488 and AlexaFluor568-conjugated goat anti-mouse or goat anti-rabbit (Life Technologies). Isotype controls (Thermo Fisher) were used for staining.

### Western Blot and Rac1 Activity Assays

Cells were washed with ice-cold PBS and lysed in RIPA buffer containing protease inhibitors for 10 min on ice. Cells lysates were cleared by centrifugation and protein levels determined (Bradford assay). Samples were boiled in laemmli buffer with ß-mercapto-ethanol for 5 min and run on Tris-glycine gels, transferred to PVDF membrane, and probed. Proteins were visualized with fluorescent secondaries (IRDye 680RD Goat anti-Mouse IgG, Li Cor, 926-68070) or chemiluminescene (HRP-linked anti-mouse IgG or anti-rabbit IgG, cell signaling, 7076S and 7974S).

Active Rac1 was measured as described previously (23). Briefly, cells were lysed (50 mM Tris-HCl (pH 7.5), 2 mM MgCl2, 0.1 M NaCl, 1% NP-40, and 10% glycerol with protease inhibitors) and incubated with Glutathione S-transferase (GST) coupled to the p21-binding domain of Pak (PBD) to precipitate Rac-GTP while rotating at 4°C. Beads were then washed 4 times in lysis buffer and resuspended in SDS sample buffer and heated (5 mins 95°C). Samples were then loaded onto SDS-PAGE and processed for Western blotting.

Protein levels were quantified using ImageJ software to measure density of target proteins bands relative to a control protein (tubulin). Results were graphed and presented as bar graphs.

### PCR

Cells were lysed in Trizol and the Zymo DirectZol kit was used for extraction of RNA and digestion of DNA according the manufacturer’s instructions. RNA levels and purity was measured using NanoDrop. cDNA was produced using qScript (Quanta Biosciences, 95048-500) and 1 µg of RNA in a total volume of 25 µl. For quantitation of gene expression exon-spanning validated FAM-labeled primers for *Rac1*, *Cdc42*, *RhoA*, *APE1*, and *18S* (Life technologies) were used and the Taqman master mix from Applied Biosystems.

### Ethical Considerations

IRB-approved, informed consent was used to obtain all human biopsy specimens.

### Confocal Microscopy

Cells were washed with and fixed in 2% formaldehyde for 15 min at 37°C. Cells were blocked and permeabilized (1.5% BSA, 5% goat serum, and 0.2% triton X-100 in PBS) for 1 h at 37°C. Antibodies (1/100) were incubated at 4°C overnight and AlexaFluor-conjugated secondary antibodies (1/500 in blocking buffer) incubated for 1 h at room temperature. AF647-conjugated Phalloidin (1/200) was added in PBS containing 1% goat serum and incubated for 20 min at room temperature. Slides were mounted in ProLong antifade Gold containing DAPI (Life Technologies). Frozen tissue section (8 µm) were fixed and stained as described for cells. Slides were imaged using an Olympus IX81 inverted confocal microscope and analyzed using Olympus FluoView 3.0 and quantified using ImageJ.

### Barrier Studies

500,000 T84 cells were seeded onto inserts (Millipore PIHA01250) and allowed to develop trans-epithelial electrical resistance (TEER of >1,000 Ω*cm^2^) (34). Trans-epithelial electrical resistance (TEER) of the cells was measured using a voltmeter (World Precision Instruments). Data is reported as relative TEER which is the TEER measurement at the experimental time point compared to start of the experiment. For infection experiments, cells were allowed to equilibrate for 1 h in Ringers solution containing 10 mM glucose. 0.1 U/ml of glucose oxidase was used to impart an oxidative stress.

### Proximity Ligation Assay

APE1-Rac1 interactions were detected using the Duolink proximity ligation assay (PLA) Kit (Olink Bioscience, Uppsala, Sweden) per the manufacturer’s protocol and as previously described (18). The PLA detects proteins by hybridizing two tags that together allow for the transcription of a fluorescent-tagged product. Briefly, cells were grown in 8-well glass chamber slides, fixed, permeabilized, and blocked as described for confocal microscopy. Rabbit-anti-APE1 (1/200) and mouse-anti-Rac1 (1/100) were used as primary antibodies and anti-rabbit PLUS and anti-mouse MINUS PLA probes (1/5) as secondary detection. Then, provided ligase-ligation solution and amplification polymerase solution were applied. Slides were mounted using supplied mounting media containing DAPI. Fluorescent signal is produced for proteins within 40 nm of each other. Quantification of relative PLA signal (red fluorescence per DAPI signal) was measured using at least nine images per condition using ImageJ software.

### Data Analysis/Statistics

Data were entered in Graphpad Prism 5 and statistical significance tested using Student’s t-test for two conditions or ANOVA with Bonferroni-corrected post-hoc testing for data with three or more different comparisons. Graphs show the mean values and error bars indicate standard errors of the mean.

## Results

### APE1 Regulates the Internalization of Bacteria Into Intestinal Epithelial Cell Lines

To select the most suitable bacteria for the studies, we first determined the ability of different bacteria to become internalized into T84 by comparing *Salmonella enterica* serovar Typhimurium (SL1344) (MOI 10), adherent-invasive *Escherichia coli* strain LF82 (AIEC/LF82), enteropathogenic *E. coli* (EPEC), *E. coli* (K12) and *C. jejuni* (all at MOI 100). CFU were evaluated following infection for 1, 2 or 4 h using the gentamicin protection assay. As expected, prolonged infection time resulted in increased internalization for all tested bacteria. Internalization was significantly lower for enteropathogenic *E. coli* (EPEC), *E. coli* K12 and *C. jejuni* (data not shown). Due to the increased ability of *S.* Typhimurium and or AIEC strain LF82 to internalize, these two species were used for all subsequent experiments.

**Figure 1:**
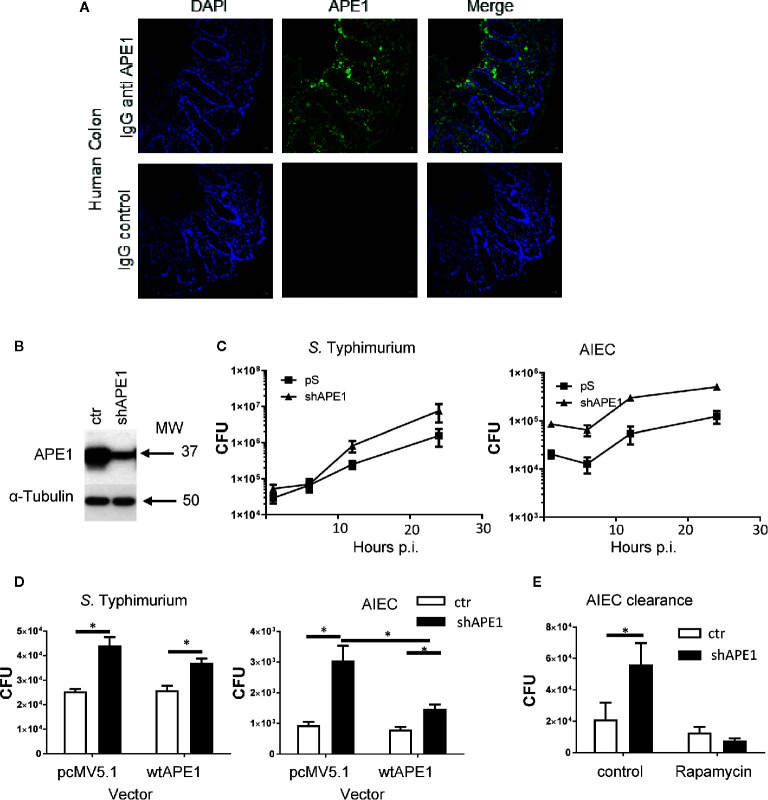
Numbers of intracellular bacteria in intestinal epithelial cells are negatively regulated by APE1. **(A)** To confirm that APE1 was expressed by human epithelial cells, human colonic biopsy specimens were stained with DAPI or goat anti-APE1 followed by an anti-goat secondary antibody and imaged. Staining can be detected in both the epithelium and lamina propria. **(B)** T84 cells containing short hairpins to APE1 have reduced levels of APE1 protein compared to cells containing a control vector. **(C)** T84 cells with normal or suppressed levels of APE1 were infected with *S.* Typhimurium at MOI 10 or AIEC at MOI 100 for 1 h and assayed for intracellular bacteria using the gentamicin inhibition assay. N= 6-8 pooled from two independent experiments. **(D)** To ensure APE1 specificity of our findings in cells containing shAPE (targeting intron), APE1 levels were complemented by transfecting HT-29 cells containing shAPE with a vector expressing wild type (wt) APE1 or a control vector (pcMV5.1) and then infected with S. Typhimurium (MOI 10) or AIEC (MOI 100) for 1 h in two independent experiments with each 3-5 replicates. Control (ctr) cells with normal levels of APE1 had lower levels of intracellular bacteria with no further reduction by the transfection to increase wild type APE1 (open bars). Inhibition of APE1 by shAPE1 (black bars) increased intracellular bacteria which decreased after complementing with wild type APE1, particularly for AIEC. **(E)** To exclude the possibility that increased intracellular bacteria were caused by impaired autophagy, T84 cells were pretreated with rapamycin before assaying for bacterial internalization (expressed as CFU) to show that APE1-deficient cells have functional autophagy. All error bars are represented as SEM. *p < 0.05

To assess the role of APE1 on internalization, we confirmed the expression of APE1 by human intestinal epithelial cells using immunofluorescence assays performed on biopsy specimens obtained from human intestine (**Figure 1A**). Subsequently, we employed cells in which APE1 expression was substantially reduced by transfecting T84 cells with short hairpin vector targeting *APE1* mRNA (shAPE1) leading to suppression of APE1 protein (**Figure 1B**). Furthermore, we specifically chose the shRNA-based APE1 knockdown method over APE1 knockout method as a previous study has shown that complete deletion of APE1 results in cellular death due to the multifunctional aspect of APE1 (35). Assays for intracellular bacteria were performed in control or APE1-deficient cells 1 h after infection to focus on the bacteria that internalized most efficiently and to minimize the effects of bacteria-induced cell death. Infection of these cells with *S.* Typhimurium or AIEC strain LF82 showed that internalization of the bacteria was increased when APE1 expression was inhibited (**Figure 1C**). To validate the observation in T84 cells, APE1 was inhibited in HT29 cells which also showed an increase in internalization (**Figure 1D**). Complementation of APE1 by transfection of pcMV5.1 APE1 into APE1-deficient cells was confirmed (**Figure S1A**) and when applied in these studies, resulted in the reduction of intracellular *S.* Typhimurium (not significantly) and AIEC numbers (p < 0.05) compared to control cells (**Figure 1D**). As numbers of increased intracellular bacteria can be caused by enhanced entry into the cells or reduced clearance of intracellular bacteria (36), autophagy function was evaluated in APE1 suppressed cells by pretreating the cells with rapamycin. Rapamycin inhibits mTOR which is a physiological inhibitor of autophagy. Treatment of cells with rapamycin allowed APE1-deficient cells to clear the majority of intracellular AIEC, indicating that APE1-deficiency did not disable the clearance of intracellular bacteria by autophagy (**Figure 1E**).

### APE1 Regulates Bacterial Internalization Into Primary Human Epithelial Cell Lines

**Figure 2:**
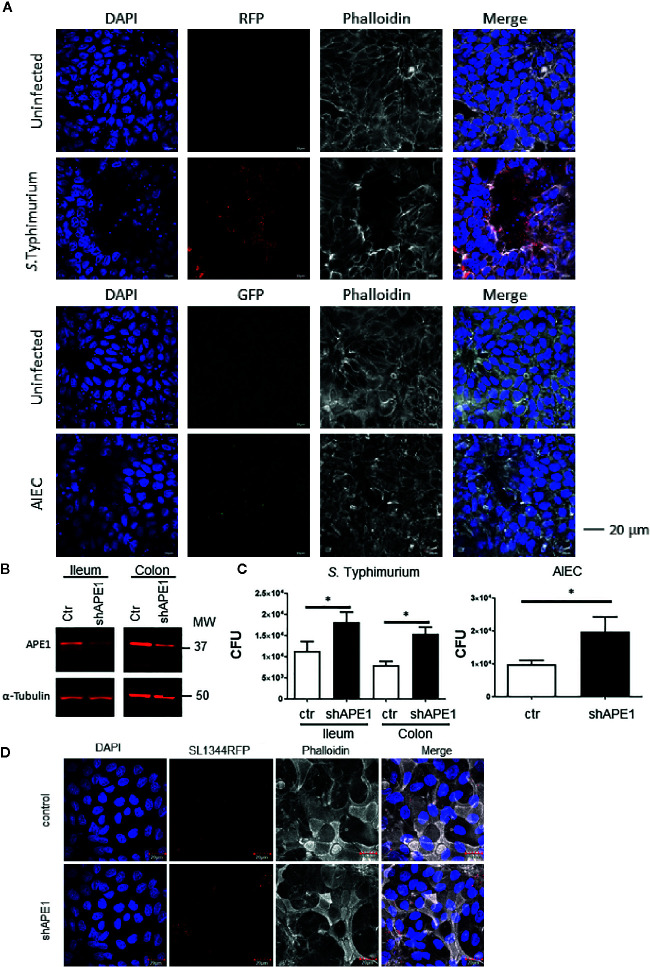
APE1 negatively regulates levels of intracellular bacteria in primary human epithelial cells. **(A)** To determine the ability of the bacteria to become internalized into human primary intestinal epithelial cells (colon), stem cells were differentiated on transwell inserts and infected for 1 h with RFP-expressing S. Typhimurium, (MOI 10) or GFP-expressing AIEC (MOI 100) and imaged by confocal microscopy. Arrows indicate clusters of S. Typhimurium, or individual AIEC. **(B)** APE1 protein levels are reduced in primary human ileal and colonic cells following transduction using lentiviruses expressing short hairpins against APE1. **(C)** Both APE1-deficient ileal and colonic primary epithelial cells show increased numbers of intracellular S. Typhimurium following infection for 1 h at MOI 10. AIEC were used to primary human ileal epithelial cells only at an MOI of 100. Numbers of intracellular bacteria were significantly increased in cells deficient in APE1. Data are shown from 5 independent experiments. **(D)** Confocal microscopic analysis confirms that APE1-deficient primary cells contain higher numbers of intracellular S. Typhimurium following infection at MOI 10. All error bars are represented as SEM. *p < 0.05

Since increased numbers of intracellular *S.* Typhimurium and AIEC strain LF82 were detected in APE1-deficient tumor cell lines, human primary epithelial cells derived from the terminal ileum or colon of healthy donors were used as a more biologically relevant target. Primary epithelial cells of ileum and colon were capable of being infected by both *S.* Typhimurium and *E. coli* strain LF82 (expressed RFP and GFP, respectively) as observed by confocal microscopy (**Figure 2A**). Subsequently, these primary epithelial cells were transduced with a lentiviral vector containing either a control shRNA or shAPE1 construct with the latter reducing APE1 expression (**Figure 2B**). Following infection, primary human APE1-deficient colonic and ileal epithelial cells showed increased numbers of intracellular *S.* Typhimurium (**Figure 2C**). As AIEC is typically observed in the ileum, primary ileal epithelial cells were infected with AIEC and tested for internalization and also showed increased numbers of intracellular bacteria when levels of APE1 were suppressed (**Figure 2C**). Confocal imaging confirmed the increase in *S.* Typhimurium when APE1 was inhibited (**Figure 2D**). Together, these data show that APE1 regulates levels of intracellular bacteria in both human cancer cell lines and primary human epithelial cells.

### Activation of Rac1 by Enteric Pathogens Is Regulated by APE1

As Rho GTPases are known to regulate bacterial internalization by invasion (14–16) or engulfment (23). To focus on a Rho GTPase that might be regulated by APE1, we evaluated which Rho GTPases were regulated by APE1 at the transcriptional level. The mRNA for three Rho GTPases (*Rac1*, *Cdc42* and *RhoA*) was assayed in T84 cells and primary human intestinal epithelial cells by qRT-PCR. *Rac1* mRNA expression was increased in APE1-deficient epithelial cells (**Figure S1B, C**) so our evaluation focused on how APE1 regulated Rac1 function in the process of bacterial internalization.

**Figure 3:**
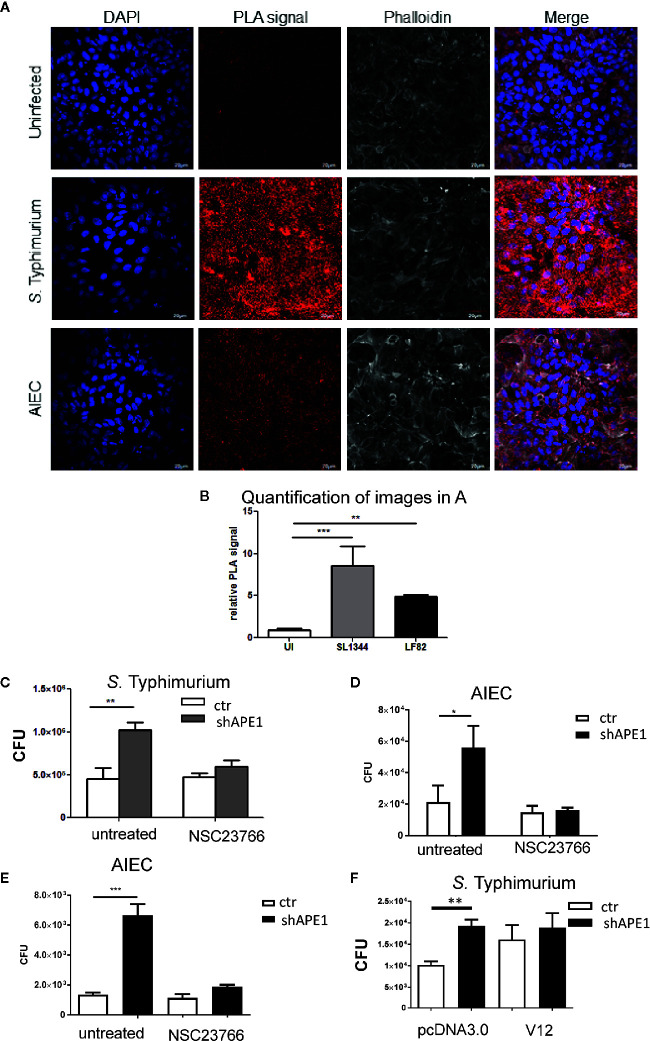
APE1 interacts with Rac1 following infection. **(A)** Primary human ileal intestinal epithelial cells were infected with S. Typhimurium (MOI 10) or AIEC (MOI 100). Following infection, cells were fixed and the co-localization of APE1 and Rac1 was analyzed by proximity ligation assay (PLA), which produces a red fluorescent signal for proteins located within 40nm of each other. Significantly increased red fluorescence showing co-localization of APE1 and Rac1 was observed following infection with S. Typhimurium and AIEC, as shown by the quantification of nine different images as shown in **(B)**. **(C)**.T84 cells were pretreated with the Rac1 inhibitor NSC 23766 overnight and then infected with S. Typhimurium at MOI 10 for 1 h. Intracellular bacteria were quantified and show reduced numbers of S. Typhimurium in APE1-deficient cells pretreated with the Rac1 inhibitor. Pre-treatment of T84 **(D)** or HT-29 **(E)** cells with the Rac1-inhibitor NSC23766 limits internalization of AIEC strain LF82. **(F)** When instead of pretreating cells with the inhibitor, HT-29 cells were transfected with active Rac1 (V12) the levels of intracellular *Salmonella* bacteria in APE1 sufficient cells became similar to the levels observed in APE1-deficient cells after a 1 h infection. All error bars are represented as SEM. *p < 0.05; **p < 0.01; ***p < 0.001

To determine that the number of intracellular bacteria was regulated through APE1-Rac1 interactions, we first established the co-localization of these molecules in human intestinal epithelial cells. Primary human ileal epithelial cells were infected with *S.* Typhimurium for 1 h and analyzed for the co-localization of APE1 and Rac1 using the proximity ligation assay (PLA). Infection of primary ileal epithelial cells with *S.* Typhimurium or AIEC increased co-localization of APE1 and Rac1 (**Figures 3A, B**). The PLA indicates a distance less than or equal to 40 nm between APE1 and Rac1. Since APE1 has been shown to physically associate with Rac1 and inhibit its contribution to the generation of reactive oxygen species (18), it is reasonable to speculate that the proximity of APE1 to Rac1 in the context of infection limits Rac1 activation and its role in the internalization of the bacteria.

As the interaction between APE1 and Rac1 may impair Rac1 activation, T84 cells or HT-29 cells were pre-treated with the Rac1 inhibitor NSC23766 overnight. NSC23766 is commonly used in multiple cell types and *in vivo* to inhibit Rac1 without effecting RhoA or Cdc42 activation (37–40). Pre-treatment of T84 cells with NSC23766 resulted in reduced numbers of intracellular *S.* Typhimurium in APE1-deficient cells thereby reversing the effect caused by the reduced APE1 expression (**Figure 3C**). Inhibition of bacterial internalization was also observed for AIEC in T84 and HT-29 cells pre-treated with NSC23766 (**Figures 3D, E**). Subsequently, HT-29 were transfected with constitutively active Rac1 or control plasmid. Control cells expressing Rac1 V12 had numbers of intracellular bacteria that were not statistically different than the numbers from APE1-deficient cells after 1 h infection (**Figure 3F**). These data showed that targeting Rac1 activation led to an intracellular bacterial burden that correlated to the effects of changing APE1 expression.

**Figure 4:**
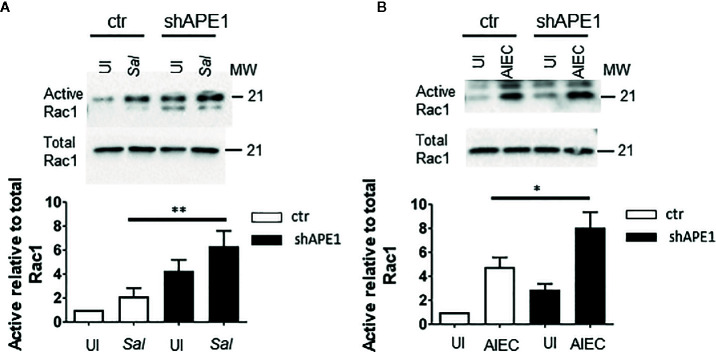
APE1 negatively regulates levels of active Rac1. **(A)** Triplicate cultures of sham (ctr) or shRNA transduced T84 cells were left uninfected (UI) or infected with S. Typhimurium. Cell lysates analyzed for levels of active GTP-bound Rac1 by a pull down assay or total Rac1. APE1-deficient cells show significantly increased levels of active Rac1 relative to total levels of Rac1 as determined by the ratios of the densitometry from four independent experiments. **(B)** As observed with S. Typhimurium, AIEC-infected APE1 deficient T84 cells show significantly higher levels of active Rac1. All error bars are represented as SEM. *p < 0.05; **p < 0.01.

As APE1 regulated the internalization of *S.* Typhimurium and AIEC and resembled the effects of manipulating Rac1 activation pharmacologically or genetically, we assessed if the inhibition of APE1 expression modulated Rac1 activation in the context of these infections. T84 cells were infected with *S.* Typhimurium or AIEC for 1 h and cell lysates were analyzed for levels of active GTP-bound Rac1 relative to total levels of Rac1. Both species of bacteria resulted in increased levels of activated Rac1 (**Figure 4**, **Figure S2**). Activation of Rac1 was significantly higher in APE1-deficient cells compared to control cells following infection with *S.* Typhimurium and AIEC (**Figure 4**). These findings suggest that APE1 impairs the activation of Rac1 and this accounts for the increase in intracellular bacteria when APE1 expression is inhibited.

### APE1 Enhances Barrier Recovery Following Infection With *Salmonella* Typhimurium

**Figure 5:**
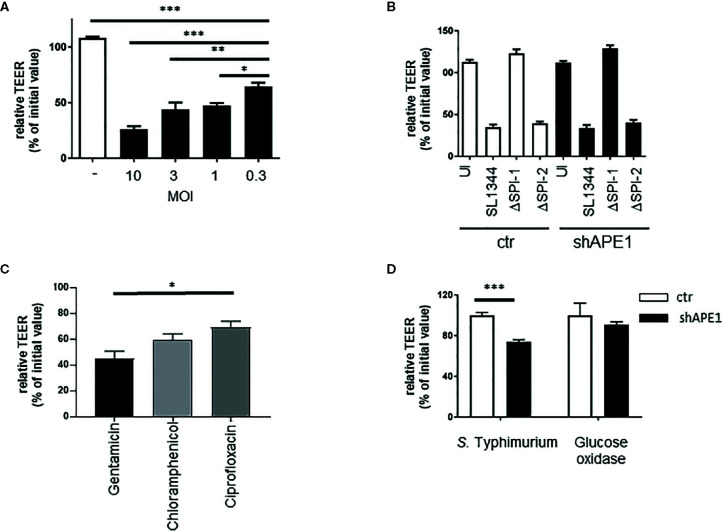
Regulation of barrier function by APE1 **A)** Compromised barrier function in T84 cells was assayed by TEER and found to be reduced with lower MOI of S. Typhimurium after 3 h (3-4 replicates). **(B)** Infection (MOI 10) for 5 h of T84 cells on transwell inserts with wild type or mutants (ΔSPI-1 or ΔSPI-2) of S. Typhimurium resulted in barrier loss by S. Typhimurium that expressed SPI-1 (wild type or ΔSPI-2) but not when SPI-1 (ΔSPI-1) was lacking as shown from four to five replicates. **(C)** To confirm the impact of the number of intracellular bacteria on barrier recovery, T84 cells were infected at MOI 10 in triplicate and treated with a cell impermeable antibiotic gentamicin, or the cell permeable antibiotics chloramphenicol or ciprofloxacin. Cell permeable antibiotic treatment resulted in improved barrier recovery and have reduced numbers of intracellular bacteria (latter data not shown). **(D)** Following infection (MOI 10) or treatment with glucose oxidase, cells were washed and cell culture medium containing gentamicin was added. Barrier recovery was recorded the next day. Data show that APE1-deficient cells infected with S. Typhimurium had reduced recovery compared to cells with similar loss in barrier induced by glucose oxidase. Data shown are from three independent experiments. All error bars are represented as SEM. *p < 0.05; **p < 0.01; ***p < 0.001.

As we observed that APE1 deficiency led to increased numbers of intracellular bacteria, we examined whether APE1 protects against barrier loss following infection with *S.* Typhimurium. Various MOI of *S.* Typhimurium (MOI 50, 25, 10) all caused a comparable decrease in TEER and dextran leakage (**Figure S3**). However, MOI lower than 10 exposed a dose-sensitive loss of barrier function (**Figure 5A**). Since this was not seen in *E. coli* (data not shown) we chose to further explore the dose-sensitive barrier function loss with *Salmonella* infection. Consistent with the reduced numbers of intracellular bacteria, *Salmonella* lacking SPI-1, which do not invade efficiently, did not result in barrier loss at MOI 10 (**Figure 5B**). Subsequently, we tested the impact of intracellular *S.* Typhimurium on the ability of the cells to recover barrier. Following infection for 1 h, TEER was assessed and cells were treated with the non-cell permeable antibiotic gentamicin or the cell permeable antibiotics ciprofloxacin or chloramphenicol. The following day, TEER was re-evaluated. Treatment of the cells overnight with cell permeable antibiotics resulted in higher barrier function recovery (**Figure 5C**). Since we observed increased numbers of intracellular *S.* Typhimurium in APE1-deficient cells, T84 monolayers were infected with *S.* Typhimurium and then treated with gentamicin. Control T84 cells were able to recover greater barrier function whereas APE1-deficient cells containing more bacteria had lower epithelial barrier function (**Figure 5D**). To confirm that APE1-deficient cells were still able to maintain and recover barrier function, cells were pulsed with glucose oxidase resulting in the generation of hydrogen peroxide (0.1 μmole/min) which is well known for compromising barrier loss (41) similar to *S.* Typhimurium infection (data not shown). After replacing the glucose oxidase with fresh medium, the glucose oxidase-treated APE1-deficient cells had barrier function similar to control cells, indicating that the inability to recover from barrier loss was caused by the infection with *S.* Typhimurium and not an effect of APE1 expression.

## Discussion

Many different pathogenic bacteria are internalized into host cells through invasion or engulfment (14–16, 23, 42–45). We found that the numbers of intracellular *S.* Typhimurium and AIEC were increased in APE1-deficient cells. Increasing numbers of intracellular *S.* Typhimurium resulted in impaired barrier function. Not only was APE1 in close proximity to Rac1, but APE1-deficient cells showed increased levels of Rac1 activation while inhibition of Rac1 activation restored the levels of intracellular bacteria to the numbers observed in control cells. Together, these data support the notion that APE1 regulates levels of intracellular *S.* Typhimurium and AIEC through its ability to inhibit the activation of Rac1 suggesting that the regulation of APE1 expression may contribute to the modulation of bacterial internalization.

Our study examined two different human cancer-derived cell lines and primary human epithelial cell cultures from different subjects, all confirming that APE1 attenuates the accumulation of intracellular bacteria. We validated the specificity of our short hairpin RNA’s by western blot analysis and showing that a plasmid encoding wildtype APE1 could restore the inhibition of intracellular bacteria by complementing shAPE1 cells (46). Our data showed that deficient autophagy ability of APE1 deficient cells was not the cause of the increased intracellular bacteria, however, further study is needed to elucidate the precise role autophagy does have in this accumulation. APE1-dependent regulation of cell internalization was observed for two different species of bacteria: *S.* Typhimurium and AIEC. We used PLA to demonstrate the co-localization of APE1 and Rac1 in primary human epithelial cells that would enable the regulatory process. In addition to evidence of proximity in the cells following infection with *S.* Typhimurium and AIEC, APE1 was able to regulate levels of active Rac1 after infection. Thus, the evidence for the involvement of Rac1 in this APE1-dependent regulation of cell-internalization by these species was based on: the co-localization of APE1 and Rac1; the inhibition of Rac1 activation by APE1; and by using both an inhibitor of Rac1 binding to guanine nucleotide exchange factors (GEF) and genetic manipulation of Rac1. The Rac1 inhibitor, NSC23376 may not be specific for Rac1 (47), however, the validation with a genetic approach and the data showing the effect of APE1 on Rac1 activation provide a compelling case that APE1 can modulate bacterial internalization, at least in part, through its effects on Rac1. While our multiple methods demonstrate the APE1 and Rac1 interaction, exactly how the interaction is initiated by bacterial infection needs to be established in future studies.

The internalization of various pathogenic bacteria into epithelial cells by means of invasion or engulfment occurs through the regulation of Rho GTPases (14, 16, 22, 23, 43–45). In particular, highly invasive *S.* Typhimurium actively regulate Rho GTPases, including Rac1 through the translocation of effector molecules (14–16, 48). Therefore, the finding that APE1 is capable of negatively regulating the levels of active Rac1 in epithelial cells would be consistent with the concept that APE1 limits Rac1-mediated internalization of bacteria. Previously, we have shown that APE1 is expressed in human gastric epithelial cells and regulates host response induced by *Helicobacter pylori* (*H. pylori*) including the activation of Rac1 and its effects on the generation of reactive oxygen species (18). However, *H. pylori* do not, in general, become internalized. The current results extend these observations to human intestinal epithelial cells as well as to the role of APE1 in the regulation of enteric pathogen internalization, thereby expanding our understanding for the multifunctional role for APE1 in bacterial pathogenesis.

An essential role of the intestinal epithelium is maintaining a barrier, which was progressively impaired with MOI increasing from 0.3 to 10. As expected, because of the increased numbers of intracellular bacteria, barrier function following infection was impaired in APE1-deficient cells. indicating that the observed differences in numbers of intracellular bacteria may impact barrier function. The impaired barrier recovery by the increased in intracellular bacteria could be reversed using cell-permeable antibiotics, but not with non-permeating antibiotics, indicating that the failure to restore barrier function was attributable to the intracellular bacteria. Thus, the regulation of bacterial internalization by APE1 may have profound effects on epithelial cell function. Future studies probing the role of APE1 in enterocytes will be enhanced by tissue-targeted knockout of the *APEX1* gene.

Initially, the function of APE1 was described in base excision repair of oxidative DNA damage, followed by its role as a redox transcriptional regulator and inhibitor of reactive oxygen species (ROS) production, including its ability to inhibit Rac1 (46, 49–51). On one hand, APE1 promotes DNA integrity by limiting levels of ROS and repairing oxidative DNA damage and on the other hand, APE1 promotes immunity by activating NF-kB and AP-1 in response to infection (18, 49, 52). The present data provide new insights into the role of APE1 in bacterial pathogenesis by showing that APE1 inhibits Rac1 activation in intestinal epithelial cells resulting in significantly lower numbers of intracellular bacteria. Mechanisms whereby APE1 negatively regulates the numbers of intracellular bacteria would complement other functions for APE1 in innate immunity to protect the host against infection. Based on the functions reported here, it is possible that the regulation of APE1 expression, or genetic polymorphisms affecting its interactions with Rac1, may affect bacterial pathogenesis. We propose that APE1 contributes to host protection at many levels including the control of bacterial burden; the accumulation of reactive oxygen species; the regulation of innate responses to help clear infection; the maintenance of the epithelial barrier; and through its role in maintaining host DNA integrity in responses to infections. Future studies should establish the importance of APE1 in host defense in a mouse model to increase our understanding of the physiological role of APE1 and potential therapeutic applications.

## Data Availability Statement

The raw data supporting the conclusions of this article will be made available by the authors, without undue reservation.

## Ethics Statement

The studies involving human participants were reviewed and approved by UCSD Institutional Review Board. The patients/participants provided their written informed consent to participate in this study.

## Author Contributions

GH and LB contributed equally in designing and performing experiments, writing text. AA provided technical support for primary cell line culture. LP collected biopsy specimens for primary cell lines. JNGA made the constructs for gene transduction. RX provided technical support. SD provided technical expertise in Rac1 assays. TS assisted in experimental design, review of data, writing of manuscript. LE assisted in experimental design, review of data, writing of manuscript. PE assisted in experimental design, review of data, writing of manuscript. SC assisted in experimental design, review of data, writing of manuscript. All authors contributed to the article and approved the submitted version.

## Funding

Funding was obtained from the National Institutes of Health (LB and LP: DK07202; SC: DK061769; PE and SC: NIH P30-DK120515; PE: AI079145; SD: DK099275) as well as the Neuroscience Microscopy Shared Facility Grant P30 (NS047101) and the Chiba University-UC San Diego Center for Mucosal Immunology, Allergy and Vaccine Development.

## Conflict of Interest

The authors declare that the research was conducted in the absence of any commercial or financial relationships that could be construed as a potential conflict of interest.

## Supplementary Material

Supplementary Figure 1
**(A)** Supplementation of wtAPE1 restored APE1 to wild type levels in shAPE knockdown HT-29 cell lines compared to control vector pcMV5.1. Control (ctr) and shAPE1 transduced T84 **(B)** or primary human epithelial cells from the ileum **(C)** were assayed for various Rho GTPases. Since APE1-deficient cells expressed increased levels of *Rac1* mRNA, it was chosen as the focus for the experiments.

Supplementary Figure 2 Additional western blots for independent experiments for *Salmonella* **(A)** and AIEC **(B)** show regulation of levels of active Rac1 by APE1 in T84 cells.

Supplementary Figure 3 Infection of T84 cells with *S.* Typhimurium at varying MOIs results in similar loss of barrier function as assayed by TEER **(A)** and paracellular leakage of FITC-dextran **(B)**.
