# Supplementary figures and images for: Apurinic/Apyrimidinic Endonuclease 1 Restricts the Internalization of Bacteria Into Human Intestinal Epithelial Cells Through the Inhibition of Rac1
Source: Front Immunol. 2021 Feb 2;11:553994. doi: 10.3389/fimmu.2020.553994 (PMC7884313; doi:10.3389/fimmu.2020.553994)

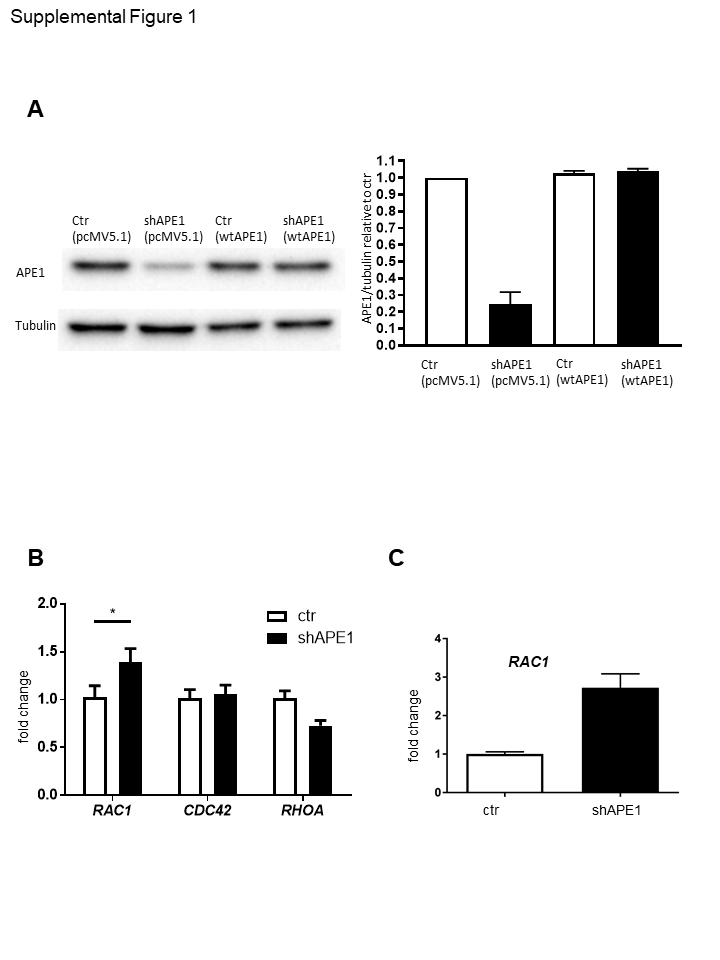

Supplement: Supplementary Figure 1 — (A) Supplementation of wtAPE1 restored APE1 to wild type levels in shAPE knockdown HT-29 cell lines compared to control vector pcMV5.1. Control (ctr) and shAPE1 transduced T84 (B) or primary human epithelial cells from the ileum (C) were assayed for various Rho GTPases. Since APE1-deficient cells expressed increased levels of Rac1 mRNA, it was chosen as the focus for the experiments. [file Image_1.tif]

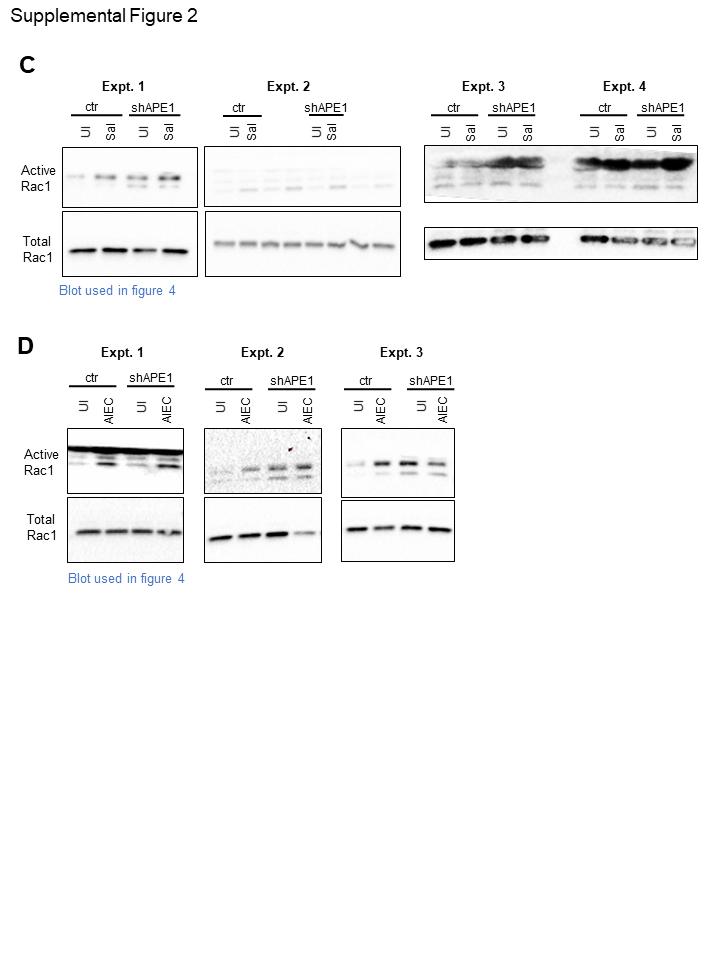

Supplement: Supplementary Figure 2 — Additional western blots for independent experiments for Salmonella (A) and AIEC (B) show regulation of levels of active Rac1 by APE1 in T84 cells. [file Image_2.tif]
